# Perforated Appendicitis with Peri-Appendicular Abscess in an Amyand’s Hernia

**Published:** 2013-10-08

**Authors:** Ruquia Khatoon, Yousuf Aziz Khan, Nasir Salim Saddal

**Affiliations:** Department of Pediatric Surgery, National Institute of Child Health (N.I.C.H), Karachi, Pakistan

**Keywords:** Amyand’s hernia, Perforated appendix, Peri-appendicular abscess

## Abstract

Amyand’s hernia is a rare clinical condition in which a normal or an inflamed appendix lies in the inguinal hernial sac. Perforated appendicitis in an Amyand’s hernia is even more uncommon. Herein we report such a rare case in a 4 month old baby who presented with an irreducible right inguino-scrotal swelling. Exploration revealed perforated appendix in the hernial sac with local abscess. A transherniotomy appendectomy was done. Postoperatively, the patient developed wound infection, which resolved with wound care. Pediatric / hernia surgeons must remember this rare clinical situation while managing children with acute right inguino-scrotal swellings.

## INTRODUCTION

Amyand’s hernia (AH) is rare variety of inguinal hernias containing vermiform appendix.[1] It is named after a Sergeant Surgeon to King George II of England, Dr. Claudius Amyand, who first described the presence of a perforated appendix in an inguinal hernia sac in an 11 years old boy in the 18th century.[1,2] AH constitutes about 1% of all cases of inguinal hernias.[3] Because of its rarity, it is seldom suspected and most of the cases are diagnosed peroperatively. Acutely inflamed appendix in an AH is extremely unusual with an incidence of only 0.1%.[1,3] Herein we report a rare case of AH with perforated appendix and peri-appendicular abscess, in an infant.

## CASE REPORT

A 4-month-old infant presented with a history of reducible right inguino-scrotal swelling since birth which had become irreducible for 4 days prior to presentation. It was associated with non-bilious vomiting and fever. On examination the child was irritable and local examination revealed an irreducible swelling in right inguino-scrotal region with redness of overlying skin and raised local temperature. Right testis was not separately appreciable due to overlying edema. Abdomen and pelvis lateral view roentgenogram showed gas filled bowel loops in the abdomen and a gas shadow in the scrotum (Fig.1). A diagnosis of strangulated inguinal hernia was initially made. After optimization of his general condition, inguino-scrotal exploration was done. About 10 cc foul smelling yellow pus was drained. The appendix was found in the hernial sac, perforated at the level of proximal third of shaft with gangrenous distal portion. The base of appendix and cecum were healthy and right testis was viable (Fig. 2). An appendectomy was performed. The stump and cecum were reduced and herniotomy done. Wound was washed thoroughly and closed primarily. Postoperative course was smooth, except for wound infection which was managed conservatively. At follow-up the infant is doing fine.

**Figure:**
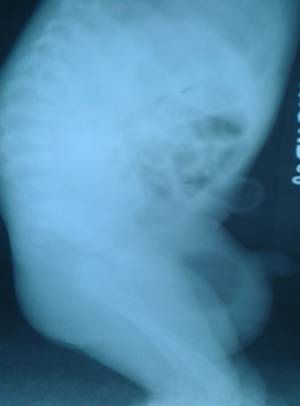
Figure 1:X-ray Abdomen and pelvis showing gas filled bowel loops and a gas shadow in the scrotum.

**Figure:**
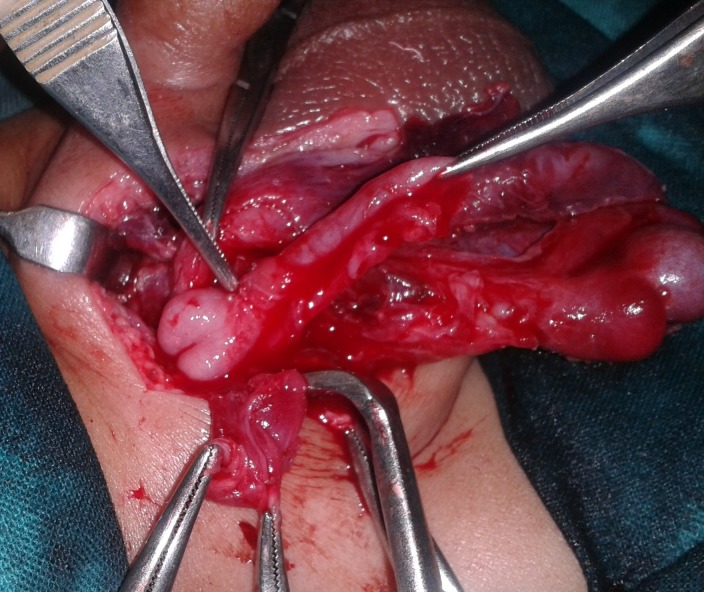
Figure 2:Per operative picture showing perforated appendix in the hernial sac.

## DISCUSSION

Finding vermiform appendix in an inguinal hernia (AH) may be an incidental finding when the patient is being electively operated for a simple inguinal hernia.[4] However, majority of the reported cases of AH have been diagnosed while exploring irreducible inguinal swellings in children with the provisional diagnosis of strangulated or obstructed inguinal hernias.[3,4] Acute or advanced stages of appendicitis in an AH are very rare with an incidence of only 0.1%.[1,5] Preoperative diagnosis is difficult. Similar situation was observed in the aforementioned case, where initial diagnosis of strangulated inguinal hernia was made.

The cause of appendicitis in an AH is not known and various hypotheses have been proposed.[6] In our case, strangulation of the herniated appendix with subsequent ischemia and necrosis could be the probable explanation. Ultrasonography and computerized tomography (CT) scan may be helpful in such cases. The clinical impression of complicated inguinal hernia is in itself an indication for surgical exploration.[3,4,6] Meinke AK recommended the use of CT scan for the evaluation of irreducible inguinal hernias and inguinal abscess, in patients at extremes of ages.[7] In our case, only plain x-ray was obtained, which showed gas shadow in the scrotum, hence, an impression of irreducible inguinal hernia was made.

There is no consensus on management protocol in cases of AH. Inflammatory status of appendix usually determines the approach.[8] In an AH with a non-inflamed appendix, only herniotomy is performed, although some recommend appendectomy especially in the cases of left sided AH.[3,4] With evidence of acute inflammation, trans-herniotomy appendectomy should be done.[1,9] In the index case, inflammation, ischemia, subsequent perforation and peri-appendicular abscess formation occurred in the portion lying outside the deep ring, therefore, only trans-herniotomy appendectomy was performed. A high mortality rate of 14-30% has been reported especially in the cases of perforated appendicitis with or without peri-appendicular abscess and peritonitis.[9]

AH with appendicitis is very rare, more so in infants. A high index of suspicion thus must be exercised. Surgeons should keep this rare condition in the list of differential diagnosis while evaluating children with irreducible inguino-scrotal swellings, and must be familiar with its management options for the optimal outcome.

## Footnotes

**Source of Support:** Nil

**Conflict of Interest:** None declared

